# Supplementary material for: Does Systemic Methotrexate Therapy Induce Azole Resistance among Endogenous Candida Strains?
Source: Antibiotics (Basel). 2021 Oct 26;10(11):1302. doi: 10.3390/antibiotics10111302 (PMC8615068; doi:10.3390/antibiotics10111302)
Supplement: Supplementary file 1 [file antibiotics-10-01302-s001.zip › antibiotics-1401528-supplementary.pdf]

antibiotics

Supplementary materials

Does Systemic Methotrexate Therapy Induce Azole Resistance among Endogenous *Candida* Strains?

Żyrek Dawid [1]*, Nowicka Joanna [1]*, Pajączkowska Magdalena [1] and Morgiel Ewa [2]

Table S1. Characteristics of samples and study participants belonging to the test group. RA- Rheumatoid arthritis, PsA- Psoriatic arthritis, SLE- systemic lupus erythematosus, AS- ankylosing spondylitis, SpA- spondyloarthritis, JIA- juvenile idiopathic arthritis, SSc- systemic sclerosis, GPA- granulomatosis with polyangiitis, SjS- Sjögren's syndrome,.

Sample	Species	MIC for Fluconazole	Gender (age), duration of MTX therapy [years], weekly dose, cause of therapy, dentures presence
T1	*C. albicans*	< 0.125 µg/mL	Female (49), 19, 20mg, RA, dentures absent
T2	*Wickerhamomyces anomalus*	< 0. 125 µg/mL	Male (47), 4, 20mg, RA, dentures absent
T3	*C. albicans*	< 0.125 µg/mL	Female (67), 1, 25mg, PsA, dentures present
T4	negative	-	Female (65), 1, 15mg, SjS, dentures present
T5	*C. albicans*	< 0.125 µg/mL	Female (42), 5, 25mg, RA, dentures absent
T6	*C. albicans*	< 0.125 µg/mL	Male (51), 6, 25mg, PsA, dentures absent
T7	*C. albicans*	0.25 µg/mL	Female (55), 2, 25mg, PsA, dentures absent
T8	*C. albicans*	< 0.125 µg/mL	Female (69), 3, 20mg, SpA, dentures present
T9	*C. albicans*	0.25 µg/mL	Female (49), 14, 20mg, PsA, dentures absent
T10	*C. albicans*	< 0.125 µg/mL	Female (49), 1, 25mg, PsA, dentures absent
T11	*1. C. krusei*<br>*2. C. glabrata*	1. < 0.125 µg/mL<br>2. 32 µg/mL	Male (77), 10, 20mg, RA, dentures present
T12	*1. C. albicans*<br>*2. C. krusei*	1. < 0.125 µg/mL<br>2. 64 µg/mL	Male (66), 15, 25mg, RA, dentures present
T13	*C. albicans*	0.25 µg/mL	Female (42), 3, 17.5mg, RA, dentures absent
T14	*C. albicans*	< 0. 125 µg/mL	Male (44), 1, 15mg, RA, dentures absent
T15	*1. C. albicans*<br>*2. C. glabrata*	1. 0.25 µg/mL<br>2. < 0. 125 µg/mL	Female (68), 3, 10mg, SLE, dentures absent
T16	negative	-	Female (61), 4, 20mg, RA, dentures absent
T17	*C. albicans*	< 0.125 µg/mL	Female (78), 5, 15mg, RA, dentures present
T18	*1. C. glabrata*<br>*2. C. albicans*	1. < 0.125 µg/mL<br>2. < 0. 125 µg/mL	Male (52), 12, 15mg, PsA, dentures absent
T19	*C. albicans*	< 0.125 µg/mL	Male (25), 7, 20mg, RA, dentures absent
T20	*C. albicans*	< 0.125 µg/mL	Female (35), 0.5, 15mg, RA, dentures absent
T21	*1. C. inconspicua*<br>*2. C. albicans*	1. 32 µg/mL<br>2. < 0.125 µg/mL	Female (29), 1.5, 25mg, SLE, dentures absent
T22	*C. albicans*	< 0.125 µg/mL	Female (33), 2, 15mg, RA, dentures absent

T23	negative	-	Male (64), 2, 25mg, GPA, dentures present
T24	*C. albicans*	< 0.125 µg/mL	Female (46), 9, 25mg, RA, dentures absent
T25	*C. albicans*	< 0.125 µg/mL	Female (28), 4, 25mg, RA, dentures absent
T26	*C. albicans*	< 0.125 µg/mL	Male (45), 3, 20mg, PsA, dentures absent
T27	negative	-	Female (70), 4, 15mg, RA, dentures present
T28	*C. albicans*	< 0.125 µg/mL	Female (63), 2, 15mg, SLE, dentures present
T29	*C. albicans*	< 0.125 µg/mL	Female (63), 5, 15mg, RA, dentures absent
T30	*C. albicans*	< 0.125 µg/mL	Male (63), 6, 10mg, RA, dentures absent
T31	*C. albicans*	< 0.125 µg/mL	Female (49), 7, 10mg, RA, dentures absent
T32	*C. albicans*	< 0.125 µg/mL	Male (57), 10, 20mg, RA, dentures absent
T33	*C. albicans*	< 0.125 µg/mL	Female (68), 4, 15mg, RA, dentures present
T34	*C. albicans*	< 0.125 µg/mL	Female (36), 13, 25mg, RA, dentures absent
T35	*C. albicans*	0.25 µg/mL	Female (57), 16, 25mg, RA, dentures absent
T36	negative	-	Male (58), 7, 25mg, RA, dentures absent
T37	*C. kefyr*	< 0.125 µg/mL	Female (55), 5, 20mg, RA, dentures present
T38	*C. albicans*	< 0.125 µg/mL	Male (43), 2.5, 12.5mg, RA, dentures absent
T39	negative	-	Female (58), 10, 25mg, RA, dentures absent
T40	*C. albicans*	< 0.125 µg/mL	Female (44), 10, 25mg, RA, dentures absent
T41	*C. krusei*	16 µg/mL	Female (66), 2, 25mg, RA, dentures present
T42	negative	-	Female (32), 14, 15mg, RA, dentures absent
T43	*C. albicans*	< 0.125 µg/mL	Male (48), 3, 10mg, RA/PsA, dentures absent
T44	*C. lusitaniae*	< 0.125 µg/mL	Male (24), 3, 7.5mg, JIA, dentures absent
T45	*C. albicans*	< 0.125 µg/mL	Female (47), 1, 15mg, SSc, dentures absent
T46	*C. albicans*	< 0.125 µg/mL	Male (65), 5, 25mg, AS, dentures absent
T47	*C. albicans*	< 0.125 µg/mL	Male (44), 8, 15mg, RA, dentures present

T48	*C. albicans*	< 0.125 µg/mL	Female (60), 8, 20mg, RA, dentures absent
T49	*1. C. krusei*<br>*2. C. krusei*	1. < 0.125 µg/mL<br>2. < 0.125µg/mL	Female (45), 6, 20mg, AS, dentures absent
T50	*C. glabrata*	< 0.125 µg/mL	Female (49), 4, 15mg, RA, dentures absent
T51	negative	-	Male (61), 4, 25mg, RA, dentures absent
T52	*C. albicans*	< 0.125 µg/mL	Female (54), 10, 15mg, RA, dentures absent

Table S2. Characteristics of samples and study participants belonging to the control group. RA- Rheumatoid arthritis, PsA- Psoriatic arthritis, SLE- systemic lupus erythematosus, AS- ankylosing spondylitis, SpA- spondyloarthritis, JIA- juvenile idiopathic arthritis, SSc- systemic sclerosis, GPA- granulomatosis with polyangiitis, SjS- Sjögren's syndrome,.

Sample	Species	MIC for Fluconazole	Gender (age), dentures presence
C1	*1. C. tropicalis*<br>*2. C. albicans*	1. 0.5 µg/mL<br>2. < 0.125 µg/mL	Male (54), dentures present
C2	*C. albicans*	< 0.125 µg/mL	Female (45), dentures absent
C3	*C. albicans*	< 0.125 µg/mL	Female (20), dentures absent
C4	negative	-	Male (25), dentures absent
C5	*C. albicans*	< 0.125 µg/mL	Female (25), dentures absent
C6	*C. albicans*	< 0.125 µg/mL	Female (50), dentures absent
C7	*C. albicans*	< 0.125 µg/mL	Female (75), dentures present
C8	*1. C. albicans*<br>*2. C. albicans*	1. 0.125 µg/mL<br>2. < 0. 125 µg/mL	Female (52), dentures absent
C9	*C. albicans*	< 0.125 µg/mL	Male (53), dentures absent
C10	*C. albicans*	< 0.125 µg/mL	Male (39), dentures absent
C11	negative	-	Male (55), dentures absent
C12	negative	-	Male (56), dentures absent
C13	negative	-	Female (70), dentures present
C14	*C. albicans*	< 0.125 µg/mL	Male (50), dentures absent
C15	*Hanseniaspora uvarum*	< 0. 125 µg/mL	Female (63), dentures absent
C16	*1. C. tropicalis*<br>*2. C. albicans*	1. 0.25 µg/mL<br>2. 0.25 µg/mL	Female (46), dentures absent
C17	*1. C. albicans*<br>*2. C. glabrata*<br>*3. C. albicans*	1. 0.25 µg/mL<br>2. 16 µg/mL<br>3. 1 µg/mL	Female (70), dentures present
C18	*1. C. tropicalis*<br>*2. C. glabrata*	1. 0.25 µg/mL<br>2. 32 µg/mL	Female (40), dentures absent
C19	*C. lusitaniae*	< 0.125 µg/mL	Female (32), dentures absent
C20	*1. C. albicans*<br>*2. Rhodotorulla mucilaginosa*	1. 0.25 µg/mL<br>2. < 0. 125 µg/mL	Female (46), dentures absent
C21	negative	-	Female (35), dentures absent
C22	negative	-	Female (46), dentures absent
C23	*1. C. albicans*<br>*2. C. dubliniensis*	1. < 0.125 µg/mL<br>2. < 0.125 µg/mL	Male (58), dentures absent
C24	*C. albicans*	0.25 µg/mL	Male (61), dentures absent
C25	*C. albicans*	0.25 µg/mL	Female (54), dentures absent

C26	negative	-	Male (46), dentures absent
C27	negative	-	Male (33), dentures absent
C28	*C. dubliniensis*	< 0.125 μg/mL	Female (57), dentures absent
C29	*C. dubliniensis*	< 0.125 μg/mL	Male (44), dentures absent
C30	*1. C. glabrata*<br>*2. C. albicans*<br>*3. C. farmata*	1. 1 μg/mL<br>2. < 0.125 μg/mL	Male (45), dentures absent
C31	*C. tropicalis*	< 0.125 μg/mL	Male (51), dentures absent
C32	negative	-	Male (50), dentures absent
C33	*C. albicans*	0.25 μg/mL	Male (49), dentures absent
C34	negative	-	Male (43), dentures absent
C35	*1. C. albicans*<br>*2. C. dubliniensis*	1. <0.125 μg/mL<br>2. < 0.125 μg/mL	Female (40), dentures absent
C36	*C. dubliniensis*	< 0.125 μg/mL	Male (36), dentures absent
C37	*C. dubliniensis*	< 0.125 μg/mL	Female (34), dentures absent
C38	*C. albicans*	< 0.125 μg/mL	Male (50), dentures absent
C39	negative	-	Female (48), dentures absent
C40	*1. C. albicans*<br>*2. C. dubliniensis*<br>*3. C. krusei*<br>*4. C. tropicalis*	1. 0.25 μg/mL<br>2. 0.25 μg/mL<br>3. 64 μg/mL<br>4. 0.25 μg/mL	Female (39), dentures absent
C41	*1. C. albicans*<br>*2. C. zeylanoides*<br>*3.C. parapsilosis*<br>*4. C. zeylanoides*	1. 0.125 μg/mL<br>2. < 0. 125 μg/mL<br>3. 0.25 μg/mL<br>4. < 0. 125 μg/mL	Male (64), dentures present
C42	*1. C. glabrata*<br>*2. C. tropicalis*<br>*3. Yarnodria lipolytica*	1. 16 μg/mL<br>2. 1 μg/mL<br>3. 64 μg/mL	Male (90), dentures present
C43	*C. albicans*	64 μg/mL	Male (76), dentures present
C44	negative	-	Male (73), dentures present
C45	negative	-	Female (77), dentures present
C46	negative	-	Female (72), dentures absent
C47	*C. albicans*	< 0.125 μg/mL	Male (74), dentures absent
C48	negative	-	Female (55), dentures absent
C49	*C.albicans*	0.25 μg/mL	Male (55), dentures absent
